# Reverse obliquity femoral neck fractures: two case reports with a literature review

**DOI:** 10.3389/fsurg.2026.1731749

**Published:** 2026-01-26

**Authors:** Zheyuan Huang, Hongjun Fu, Haoyuan Liu, Xiaolin Chen, Jianming Huang

**Affiliations:** 1 Department of Orthopedics, The 73st Group Army Hospital of PLA, Xiamen, Fujian, China; 2 Department of Orthopedics, Xiamen University Affiliated Chenggong Hospital, Xiamen, Fujian, China; 3 Department of Orthopedics, Xiangyang Hospital of Traditional Chinese Medicine, Xiangyang, Hubei, China

**Keywords:** case report, femoral neck fracture, internal fixation, Pauwels angle, reverse obliquity

## Abstract

**Introduction:**

Vertical femoral neck fractures—those with a Pauwels angle >70°—are an especially demanding subset, notorious for their recalcitrance to fixation and high risk of non-union. We propose a previously unrecognized variant of femoral neck fracture in which the fracture plane is vertical (Pauwels ≥ 90°) and lacks any Gotfried-positive cortical contact, a configuration we designate “reverse-obliquity femoral neck fracture” (ROFNF). This report describes two cases of ROFNF and their respective therapeutic strategies.

**Case report:**

The index patient was a 56-year-old woman who sustained a Pauwels-III femoral neck fracture (95°) after slipping while playing table tennis. The second patient, a 45-year-old male, sustained a right femoral neck fracture (Pauwels III, 90°) during an electric vehicle rollover. Lumbar epidural anesthesia was administered supine on a fluoroscopy-compatible table for both cases. Following unsuccessful closed anatomical reduction, an anterior hip approach was utilized for open reduction. Fixation consisted of three 7.3 mm cannulated screws supplemented by a medial buttress plate; radiographs at 8–9 months confirmed uneventful union in both patients.

**Conclusion:**

We were unable to find any prior description of a femoral-neck fracture whose inclination reaches or exceeds 90° while also failing every Gotfried cortical-support criterion. In the two patients presented, closed manipulation could not restore a stable reduction; instead, an anterior approach with open reduction and a screw-plus-medial-buttress construct produced solid union.

## Introduction

Femoral neck fractures continue to pose a surgical challenge because reduction is difficult, fixation often fails, and both nonunion and osteonecrosis of the femoral head remain common (1). The Pauwels classification system, a biomechanically relevant framework for femoral neck fractures, categorizes these injuries based on the angle between the fracture line and the horizontal plane (Pauwels angle): type I (<30°), type II (30°–50°), and type III (>50°) (2). Among these, Pauwels type III fractures are particularly problematic due to excessive shear forces at the fracture site, predisposing to postoperative coxa vara and high internal fixation failure rates (3).

Clinically, we have identified a distinct subset of femoral neck fractures characterized by a Pauwels angle ≥90° and the absence of Gotfried-positive support—defined as uninterrupted cortical continuity at the fracture interface, a critical radiological marker of fracture stability (4). Herein, we term this entity “reverse obliquity femoral neck fracture (ROFNF)”. Given its extreme Pauwels angle (≥90°) and lack of cortical support, ROFNF is logically inferred to exhibit greater biomechanical instability, a higher propensity for complications, and potentially poorer clinical outcomes compared to typical Pauwels type III fractures (>50° but <90°). This deduction is based on the well-established biomechanical principle that shear forces at the fracture site increase exponentially with the Pauwels angle, while the absence of Gotfried-positive support eliminates the inherent bony stability required for successful internal fixation (3, 4).

To date, no prior literature has described such fractures. We present two cases of ROFNF with a Pauwels angle ≥90°, aiming to provide clinical insights for managing this rare and challenging entity.

## Case presentation

Written informed consent was secured from both participants prior to publication.

## Case 1

**Figure 1:**
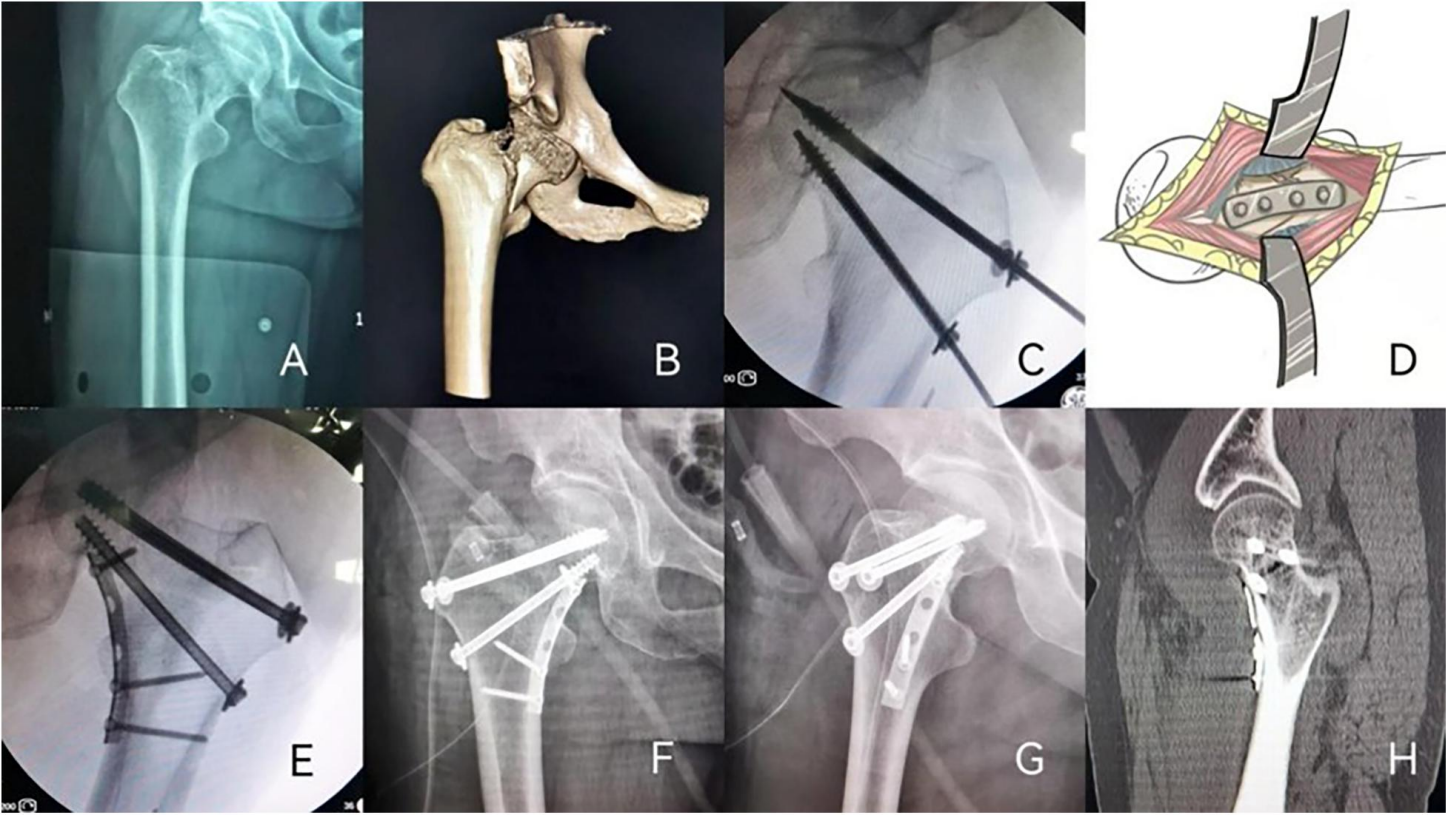
A 56-year-old female patient with right reverse obliquity femoral neck fracture who was treated by cannulated screw (CS), medial buttress plate (MBP). **(A,B)** Preoperative x-rays examination and CT scan suggested a fracture of the right femoral neck (Pauwels III-type, Pauwels angle was 95°); **(C–E)** the patient was treated with CS, MBP. Excellent reduction was achieved; **(D)** The illustration of extra exposure of femoral neck fracture and fixation by medial buttress plate. The femoral neck fracture was exposed through an anterior hip approach. **(F–H)** the x-rays examination and CT scan after 9 months postoperative follow-up showed fracture healing, no femoral neck shortening, and no implant failure.

A 56-year-old female (height 160 cm, weight 60 kg, BMI 23.4 kg/m^2^) presented with right hip pain and limited mobility 18 h after a slip during table tennis. While attempting to return a serve during a table tennis match, Patient fell due to an uncontrolled twisting of the upper torso, landing with the right hip in external rotation and sustaining the injury. Preoperative right hip x-rays and CT scans confirmed a femoral neck fracture classified as Pauwels type III with a Pauwels angle of 95° (Figures 1A,B). The patient denied any history of tobacco use or alcohol intake. This case was not associated with injuries to other anatomical regions.

### Surgical management

Preoperative antibiotic prophylaxis with 1.5 g intravenous cefuroxime was given 30 min prior to surgery. Positioned supine on a fluoroscopy-compatible table under lumbar epidural anesthesia, the patient first underwent attempted closed anatomical reduction. This proved unsuccessful owing to profound instability. Subsequently, access was then gained anteriorly by slipping through the interval between tensor fasciae latae and sartorius; the deep fascia was opened along the medial edge of the tensor to complete the window. Joint capsule exposure was then achieved through the plane between the rectus femoris and gluteus medius. The fracture was openly reduced and stabilized with three 7.3 mm cannulated screws; the cephalic pair was deliberately angled to meet the fracture plane at right angles, a position verified on fluoroscopy. After gentle hip flexion and external rotation of the injured limb, a 5-hole 1/3 tubular plate (DOUBLE, China) was contoured and fixed to the medial femoral cortex as a buttress (Figures 1C–E). Wound closure was performed in layers after fluoroscopic confirmation of adequate fixation. Postoperative antibiotic prophylaxis consisted of intravenous cefuroxime 1.5 g every 12 h for 48 h. Low molecular weight heparin (6 kD per day) and a lower extremity venous pump were used to prevent deep venous thrombosis in lower extremities. Admission to the intensive care unit was not required for this patient in the preoperative or postoperative period. The length of hospital stay for this patient was 12 days. No surgical site infection or other postoperative complications were observed in this case.

### Follow-up

Serial follow-ups at 1 week, 1, 2, 3, 6, and 9 months postoperatively showed progressive healing. At 9 months, radiographs and CT scans confirmed complete bony union without femoral neck shortening or implant failure (Figures 1F–H). Postoperative function reached excellent levels: Visual Analogue Scale (VAS) confirmed pain-free state (0/10), Short Form-36 (SF-36) indicated moderate health status (40/100), while Harris Hip Score demonstrated near-full recovery (91/100).

## Case 2

**Figure 2:**
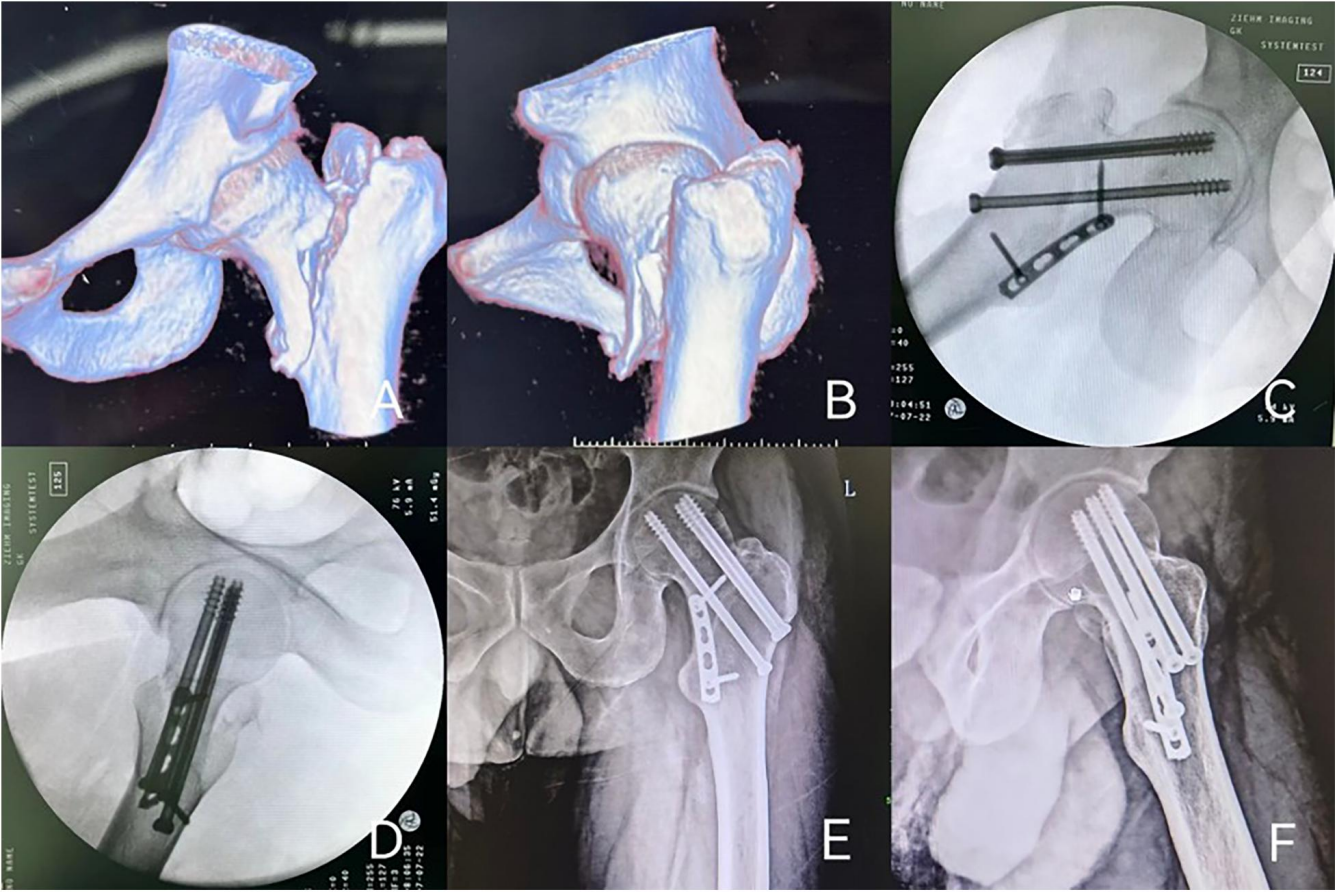
A 45-year-old female patient with left reverse obliquity femoral neck fracture who was treated by cannulated screw (CS), medial buttress plate (MBP). **(A,B)** Preoperative CT scan suggested a fracture of the left femoral neck (Pauwels III-type, Pauwels angle was 90°); **(C,D)** the patient was treated with CS, MBP. Excellent reduction was achieved; **(E,F)** the x-rays examination after 8 months postoperative follow-up showed fracture healing, no femoral neck shortening, and no implant failure.

A 45-year-old man (height 174 cm, weight 73 kg, BMI 24.1 kg/m^2^) presented with left hip pain and immobility 3 h after an electric vehicle rollover. The patient sustained a left hip injury due to external rotation and torsion forces during a rollover motor vehicle collision. The collision occurred when the patient, who was driving an electric vehicle, swerved to avoid a bicyclist traveling in the wrong direction. CT before surgery showed a vertical fracture of the left femoral neck with a 90° inclination, meeting Pauwels type III criteria (Figures 2A,B). The patient had a 25-year smoking history (10 cigarettes/day) and 23-year alcohol consumption history (150 mL white wine/day). This case was not associated with injuries to other anatomical regions.

### Surgical management

Similar to Case 1, closed reduction failed, prompting open reduction via an anterior hip approach. Because the Pauwels angle was modest, three screws were placed in the familiar upside-down triangle. A trimmed distal radius plate (DOUBLE, China) was used as the medial buttress plate (Figures 2C,D), with fixation confirmed fluoroscopically. Postoperative antibiotic prophylaxis consisted of intravenous cefuroxime 1.5 g every 12 h for 48 h. Low molecular weight heparin (6 kD per day) and a lower extremity venous pump were used to prevent deep venous thrombosis in lower extremities. Admission to the intensive care unit was not required for this patient in the preoperative or postoperative period. The length of hospital stay for this patient was 10 days. No surgical site infection or other postoperative complications were observed in this case.

### Follow-up

Follow-ups at 1 week, 1, 2, 3, 6, and 8 months postoperatively demonstrated healing. At 8 months, radiographs confirmed complete union without shortening or implant failure (Figures 2E,F). Functional outcomes were excellent: VAS score 0, SF-36 score 41, and Harris Hip Score 93.

## Discussion

Originating in 1935, the Pauwels classification categorizes femoral neck fractures according to the Pauwels angle—a radiographic measurement defined by the angle subtended between the fracture plane and the inter-anterior superior iliac spine line. Within this framework, Pauwels type III fractures (angle >50°) are characterized by high shear forces at the fracture site, exhibiting a pronounced tendency toward hip varus. This biomechanical feature predisposes to fracture displacement, varus collapse, and frequent failure of conventional internal fixation with three inverted triangular cannulated screws, often necessitating adjunctive procedures such as external rotation osteotomy (5). Consensus exists among researchers that early surgical intervention is critical to optimize outcomes in such cases (6, 7).

Notably, a distinct subset of femoral neck fractures exists, characterized by a Pauwels angle ≥90° and a complete absence of Gotfried-positive support (a key marker of fracture stability defined by continuous cortical apposition along the fracture line). This entity, which has not been previously described in the literature, we term ROFNF—a designation reflecting its unique biomechanical profile of extreme shear forces. To the best of our knowledge, these cases constitute the first documented occurrence of ROFNF in medical literature.

From a biomechanical perspective, vertically oriented femoral neck fractures (characterized by elevated Pauwels angles) experience intensified shear stresses, elevating the probability of non-union and implant failure (8). Frank Liporace et al. further refined this concept by defining “vertical femoral neck fractures” as those with a shear angle >70° (9), underscoring their heightened instability. Jiang et al. reviewed 755 femoral neck fractures with ≥2-year follow-up and showed that greater inclination in both vertical and oblique planes is linked to poorer interfragmentary stability and higher reoperation rates (10). Nevertheless, their cohort excluded fractures manifesting complete axial discontinuity between proximal and distal fragments—the defining characteristic of ROFNF—thereby providing biomechanical rationale for the heightened instability and poorer prognosis associated with ROFNF compared to other high-shear fracture variants.

Closed reduction with three cannulated screws remains the most widely used treatment for femoral neck fractures, but its efficacy in high-shear scenarios is limited (11). Zhang et al. tracked 54 vertical femoral neck fractures fixed with three parallel cannulated screws and recorded a 41% mechanical failure rate: 19 screws backed out, 18 necks shortened, 14 drifted into varus, and 8 redisplaced (12). These findings highlight the need for augmented fixation strategies in unstable fractures.

To counter this, several authors now supplement the screw construct with a medial buttress plate delivered through an anterior interval, reporting encouraging early results (7, 13). Finite-element work supports the strategy: Zhan et al. built five Pauwels-70° fracture models and compared three cannulated screws alone with the same screws plus a medial locking plate. Their analysis of seven biomechanical parameters (including construct stiffness) demonstrated that the buttress plate improves mechanical stability, enhances shear resistance, and mitigates varus collapse. Notably, the inclusion of a proximal screw in the buttress plate emerged as a critical technical detail, augmenting anti-shear capacity, reducing stress on cannulated screws, and preventing fracture redisplacement (14).

Consistent with these findings, both cases in our series were managed with a medial femoral neck buttress plate (incorporating proximal screws) to counteract extreme shear forces. With no purpose-built neck buttress available, we followed earlier reports and contoured a 1/3 tubular plate or a shortened distal-radius plate to fit. The limited cohort size precluded meaningful comparative analysis between the two plate configurations (7, 15).

Recent studies have also highlighted the utility of off-axis screws in managing high-shear femoral neck fractures (16, 17). Orienting the screws at right angles to the fracture plane boosts anti-slip stability; we therefore used this tactic in case 1, whose Pauwels angle was the larger.

Based on our management of these cases, open reduction through an anterolateral surgical access augmented by medial buttress plating and triple cannulated screw fixation demonstrated favorable mid-term functional and radiographic results for ROFNF. Based on our observations, we propose the following clinical characteristics of this fracture type: (1) High-energy traumatic mechanisms are typically implicated; (2) The fracture line descends obliquely from the proximal fragment, precluding Gotfried-positive support under gravitational loading; (3) Anatomical reduction is seldom attainable through closed methods, mandating open surgical intervention; (4) Excessive shear forces at the fracture interface predispose patients to varus hip deformity and implant failure; (5) Suboptimal therapeutic management correlates with significantly higher postoperative disability rates. Radiographic diagnostic criteria for ROFNF include: (1) Pauwels angle ≥90°; (2) Fracture plane continuity from femoral head through neck to base on anteroposterior projection; (3) Lateral view demonstrating three distinct morphological patterns: (a) Cephalic fragment encompassing head and anterior neck, with fracture cleavage connecting posterosuperior head to anterior-inferior neck base; (b) Proximal fragment containing head and posterior neck, exhibiting fracture trajectory from anterosuperior head to posteroinferior neck base; (c) The proximal fragment comprises the femoral head and medial neck, with the fracture line running from the head to the medial base of the neck.

## Limitation

This study has limitations, primarily the small sample size (*n* = 2), which precluded evaluation of all potential treatment modalities. Future studies with larger cohorts are necessary to validate our proposed clinical and radiological characteristics of ROFNF and refine evidence-based management algorithms.

## Conclusion

The two cases of ROFNF reported herein represent one of the most challenging subtypes of femoral neck fractures to manage. Our findings indicate that closed reduction and stable fixation are particularly difficult in this setting, whereas open reduction via an anterior hip approach with combined fixation (cannulated screws + medial buttress plate) offers a viable treatment strategy.

## Data Availability

The raw data supporting the conclusions of this article will be made available by the authors, without undue reservation.
